# Short-term outcomes and mortality predictors in 41 dogs undergoing cholecystectomy for gallbladder mucocele: a single referral centre study

**DOI:** 10.1186/s12917-026-05648-9

**Published:** 2026-06-22

**Authors:** G. Pagani, E. I. Ferraris, V. Montinaro, P. Buracco, G. Capra, E. Mazzone, M. Olimpo

**Affiliations:** 1 Clinica Veterinaria Malpensa-AniCura, Via Guglielmo Marconi 27, Samarate, 21017 VA Italy; 2 Department of Veterinary Science, University of Turin, Largo P. Braccini 2-5, Grugliasco (Turin), TO 10095 Italy

**Keywords:** Gallbladder mucocele, Leukopenia, C reactive protein, Abdominal effusion, Necrotising cholecystitis

## Abstract

**Background:**

Gallbladder mucocele (GBM) in dogs is a challenging disease associated with variable surgical outcomes. Several prognostic factors have been proposed, but predictors of short-term mortality remain incompletely defined. This retrospective study aimed to report the 30-day mortality rate following cholecystectomy for GBM and to evaluate potential prognostic factors in a population of client-owned dogs treated at a referral centre between 2016 and 2022.

**Results:**

Forty-one dogs met the inclusion criteria. The overall 30-day mortality rate was 14.6% (6/41). Factors significantly associated with mortality included preoperative leukopenia (*P* = 0.01), abdominal effusion detected on preoperative ultrasonography (*P* = 0.05), and necrotizing cholecystitis, diagnosed on histopathology (*P* = 0.05). Elevated C-reactive protein (> 0.8 mg/dL) was associated with higher 30-day mortality compared to normal CRP (3/6 [50.0%] vs. 7/35 [20.0%]; *P* = 0.01), as were leukopenia (3/6 [50.0%] vs. 9/35 [25.7%]; *P* = 0.01), abdominal effusion (5/6 [83.3%] vs. 26/35 [74.3%]; *P* = 0.05), and necrotizing cholecystitis (3/6 [50.0%] vs. 7/35 [20.0%]; *P* = 0.05).

**Conclusions:**

Leukopenia, increased C-reactive protein, and abdominal effusion are preoperatively identifiable predictors of poor outcome following cholecystectomy for GBM in dogs, and their early recognition may improve risk stratification and perioperative decision-making. Necrotizing cholecystitis was also identified as a histopathological predictor of mortality; while this finding is not available in time to influence postoperative management, it represents an interesting prognostic observation. These findings should be interpreted cautiously due to the retrospective design and limited sample size.

## Background

Gallbladder mucocele (GBM) is characterized by the accumulation of bile, sludge, and mucus within the gallbladder, leading to impaired excretion through the common bile duct [1, 2]. Chronic GBM may result in gallbladder overdistension and intrahepatic bile duct dilatation, increasing the risk of biliary duct obstruction and peritonitis [3]. The etiopathogenesis of GBM remains unclear, though several predisposing factors have been reported. Shetland Sheepdogs, Cocker Spaniels, and Miniature Schnauzers may be characterized by a higher risk, but other breeds may also be affected. Additional risk factors have been identified in endocrinopathies (specifically hyperadrenocorticism, hypothyroidism, diabetes mellitus), and hyperlipidemia [2, 4–6]. Clinical signs are often nonspecific, including vomiting, anorexia, and lethargy [7]. In severe cases, GBM can progress to gallbladder rupture due to transmural ischemic necrosis [3, 7–9]. Histopathologically, GBM is defined by cystic mucinous hyperplasia of the gallbladder epithelium, which underlies the progressive accumulation of abnormal mucus and biliary sludge [8]. In advanced or complicated cases, additional findings may be present. Ischaemic necrosis, most frequently affecting the fundus, can occur as a consequence of progressive intraluminal distension compromising mural perfusion, and may ultimately lead to gallbladder rupture [9]. It is important to distinguish this mechanism from rupture secondary to extrahepatic biliary obstruction, in which increased intraluminal pressure results in biliary tree disruption, these represent overlapping yet pathophysiologically distinct entities in the context of GBM [3, 6] Necrotizing cholecystitis secondary to extrahepatic bile duct obstruction has also been reported [3, 6]. The mortality rate in such cases is up to 45% [10], attributed to complications including anaemia, hypoproteinaemia, disseminated intravascular coagulation (DIC), aspiration pneumonia, thromboembolism, sepsis, and pancreatitis [11, 12]. Cholecystectomy performed either via median laparotomy (open surgery) or laparoscopy is considered the gold standard for elective GBM [13, 14]. Catheterisation of the common bile duct is sometimes performed to evaluate its patency. However, dogs undergoing cholecystectomy without common bile duct catheterisation are generally characterized by an improved long-term outcome, with reduced morbidity and mortality, compared with those in which common bile duct catheterisation is performed [7, 15]. In particular, common bile duct catheterisation has been associated with postoperative pancreatitis because of possible reflux of bile into the pancreatic duct or trauma to the major duodenal papilla [16]. Finally, retrograde catheterisation has been associated with persistent postoperative gastrointestinal signs compared to normograde techniques [17]. Historically, surgery for GBM carried high mortality rates, ranging from 20% to as high as 60% in cases of biliary peritonitis [1, 3, 7]. However, more recent studies have shown improved survival, with reported mortality rates of 2–6% for elective cholecystectomy and 20–23% for emergency procedures [12, 18] Reported postoperative mortality risk factors include hypotension, dyspnea, hypoalbuminemia, elevated bilirubin, and increased band neutrophil percentages [19, 20]. In a retrospective study including dogs undergoing biliary procedures (including cholecystectomy), 29% died within 30 days postoperatively [19]. Prognostic variables that have been associated with increased perioperative mortality include gallbladder mucocele type on ultrasound examination, serum creatinine and phosphorus concentrations, concurrent hyperadrenocorticism, hyperbilirubinemia, and Pomeranian breed [13, 21]. Additionally, C-reactive protein (CRP) level has been recognized as an indicator of gallbladder rupture [22, 23].

The present study aims to review the prognosticators and report the mortality rate within 30 postoperative days in a series of dogs undergoing surgery for GBM at a single referral center.

## Methods

Electronic medical records relative to dogs surgically treated for GBM at a single referral centre (Clinica Veterinaria Malpensa-AniCura, Italy) between January 2016 and December 2022 were retrieved. Dogs were included if they underwent cholecystectomy for GBM and met the following criteria: (a) preoperative abdominal ultrasound examination suggestive of GBM, with grading based on Choi et al. (2014) ultrasonographic classification system [24]; (b) preoperative work-up including complete blood count (CBC) and serum biochemistry panel; (c) complete surgical report with intraoperative macroscopic evidence of GBM; (d) bacteriological sampling of bile for aerobic bacterial culture and antibiotic sensitivity testing; (e) histological evaluation of the gallbladder; and (f) follow-up data for at least 30 postoperative days. Dogs were excluded if medical records were incomplete regarding any of the above parameters, if GBM diagnosis was not confirmed histologically, or if the follow-up at 30 days was unavailable.

Data retrieved included signalment (breed, sex, age, and weight), concurrent diseases, type, and duration of clinical signs, results of preoperative bloodwork, and abdominal ultrasound examination findings (including the presence of abdominal effusion and/or focal peritonitis). GBM grading was recorded according to Choi’s classification system [24] (type 1, immobile echogenic bile; type 2, incomplete stellate pattern; type 3, typical stellate pattern; type 4, kiwi like pattern and stellate combination; type 5, kiwi like pattern with residual central echogenic bile; and type 6, kiwi like pattern). Previous medical treatments, including antimicrobial administration, were also recorded. To assess the preoperative status of each dog, attention was focused on parameters such as leukocytosis (WBC > 17 × 10³ cells/µL) or leukopenia (WBC < 5.6 × 10³ cells/µL), anemia (PCV < 35%), serum biochemical parameters (ALP, ALT, GGT, albumin, BUN, creatinine, bilirubin, CRP), and ASA status. Intraoperative findings noted from the surgical report included, the procedure and type of choledochal catheterisation (if performed), intraoperative complications, and overall surgical duration. Hypotensive episodes during anesthesia (intended as mean arterial pressure [MAP] < 60 mmHg), if any, were recorded. Additionally, results of the histopathology of gallbladder and liver (if performed) and bile bacteriological cultures were included.

Postoperative complications occurring within 30 days from surgery were recorded. The 30-day mortality rate was calculated as the proportion of dogs that died or were euthanized within 30 postoperative days. For the purpose of this study, 30-day mortality was defined as death or euthanasia occurring within 30 postoperative days due to any cause related to the surgical condition, postoperative complications, or deterioration of concurrent systemic disease. No deaths attributable to causes clearly unrelated to the underlying condition or its management were recorded in this cohort.

Preoperative stabilisation was performed in all dogs that were not deemed stable prior to surgery, with regard particularly to hemodynamic status, blood pressure, or hydration status. Stabilisation was considered achieved when patients had adequate perfusion parameters (including normal mucous membrane colour and capillary refill time < 2 s), systolic blood pressure > 90 mmHg or MAP ≥ 65 mmHg, and sufficient correction of dehydration. Broad-spectrum antimicrobial therapy was initiated in dogs with suspected infection or systemic inflammatory response.

In dogs with concurrent diseases (endocrine, metabolic, hemodynamic), additional measures were undertaken if required with the goal to reach an early and sufficient to adequate presurgical stabilisation, especially in urgent cases.

All dogs were premedicated with methadone (0.2–0.3 mg/kg IM, Semfortan Dechra) combined with acepromazine (0.02–0.05 mg/kg IM, Prequilan Fatro SpA) in haemodynamically stable patients, or with methadone alone in haemodynamically compromised patients (ASA 4). Anaesthesia was induced with propofol (2–6 mg/kg IV to effect, Proposure Boehringer Ingelheim Animal Health Italia) or alfaxalone (1–3 mg/kg IV to effect, Alfaxan Zoetis) and maintained with isoflurane in oxygen. Continuous rate infusions of fentanyl (2–5 µg/kg/h, Fentadon Dechra) and ketamine (0.1–0.6 mg/kg/h, Nimatek Dechra) were administered intraoperatively for analgesia and haemodynamic support. Dopamine (5–10 µg/kg/min IV, Dopamina Pfizer) or noradrenaline (0.1–0.3 µg/kg/min IV, Noradrenalina Gelenica Senese) were administered in the event of intraoperative hypotension (MAP < 60 mmHg). Standard intraoperative monitoring included electrocardiography, pulse oximetry, capnography, invasive or oscillometric blood pressure, and body temperature. Crystalloid fluid therapy (lactated Ringer’s solution, 5–10 mL/kg/h, Braun Vet Care) was administered intraoperatively in all cases.

Statistical analysis was performed using R software (version 4.3.1, R Core Team, Vienna, Austria). Descriptive statistics were calculated for all variables. Continuous variables were reported as median and range or interquartile range (IQR) when appropriate due to non-normal distribution. Categorical variables were reported as frequencies and percentages. Key variables of interest were analyzed using Fisher’s exact test for categorical data to identify factors associated with 30-day mortality. Adjusted p-values were calculated using the false discovery rate (FDR) method to account for multiple comparisons. Results were considered statistically significant if the adjusted p-value was 0.05 or less. Findings with an unadjusted p-value of 0.05 or less but adjusted p-value greater than 0.05 were also noted as potentially clinically relevant trends. Given the retrospective nature of this study, no a priori sample size calculation was performed, and all eligible cases within the study period were included.

## Results

A total of 45 dogs surgically treated for GBM were initially identified from the database, but 4 dogs were excluded due to incomplete medical records, resulting in 41 dogs for final analysis. The cohort comprised 21 males (13 neutered, 63.2%) and 20 females (8 neutered, 40%), median age was 11 years (range, 5–16 years) and median body weight was 11.5 kg (range, 2.7–35 kg). Purebred dogs predominated (33/41, 80.5%), with mixed-breed dogs accounting for the remaining cases (8/41, 19.5%). The most represented breeds included Chihuahua (6/41, 14.6%), Maltese (4/41, 9.8%), and Pug (3/41, 7.3%). At presentation, clinical signs were recorded for all dogs. The most frequently observed were lethargy (19/41, 46.3%), vomiting (15/41, 36.6%), abdominal pain (13/41, 31.7%), and inappetence (10/41, 24.4%). Icterus was noted in 3 dogs (7.3%). Less common clinical signs included hematuria, coughing, polyuria and polydipsia, and tremors, each observed in 3 dogs (7.3%).

Abdominal ultrasonography was suggestive of GBM in all 41 dogs. Additional ultrasonographic findings included focal peritonitis surrounding the gallbladder in 32 dogs (78%), extrahepatic biliary dilation or suspected obstruction in 25 dogs (61%), abdominal effusion in 7 dogs (17.1%), and suspected gallbladder rupture in 5 dogs (12.2%). Grading of GBM based on the Choi’s 6-grade system yielded the following distribution: grade 1 in 10 dogs (24.4%), grade 2 in 19 dogs (46.3%), grade 3 in 3 dogs (7.3%), grade 4 in 3 dogs (7.3%), grade 5 in 5 dogs (12.2%), and grade 6 in 1 dog (2.4%).

Results of the preoperative hematological and biochemical evaluations are reported in Table 1. Leukocytosis (WBC > 17.0 × 10³ cells/µL) was observed in 19/41 (46.3%) dogs, leukopenia (WBC < 5.6 × 10³ cells/µL) in 6/41 (14.6%) dogs, and anemia (PCV < 35%) in 2/41 (4.9%) dogs. Elevated CRP (> 0.8 mg/dL) was found in 8/41 (19.5%) dogs. Based on American Society of Anesthesiologists (ASA) scores, 10 dogs were categorized as ASA 2, 22 dogs as ASA 3, and 9 as ASA 4.

**Table 1:**
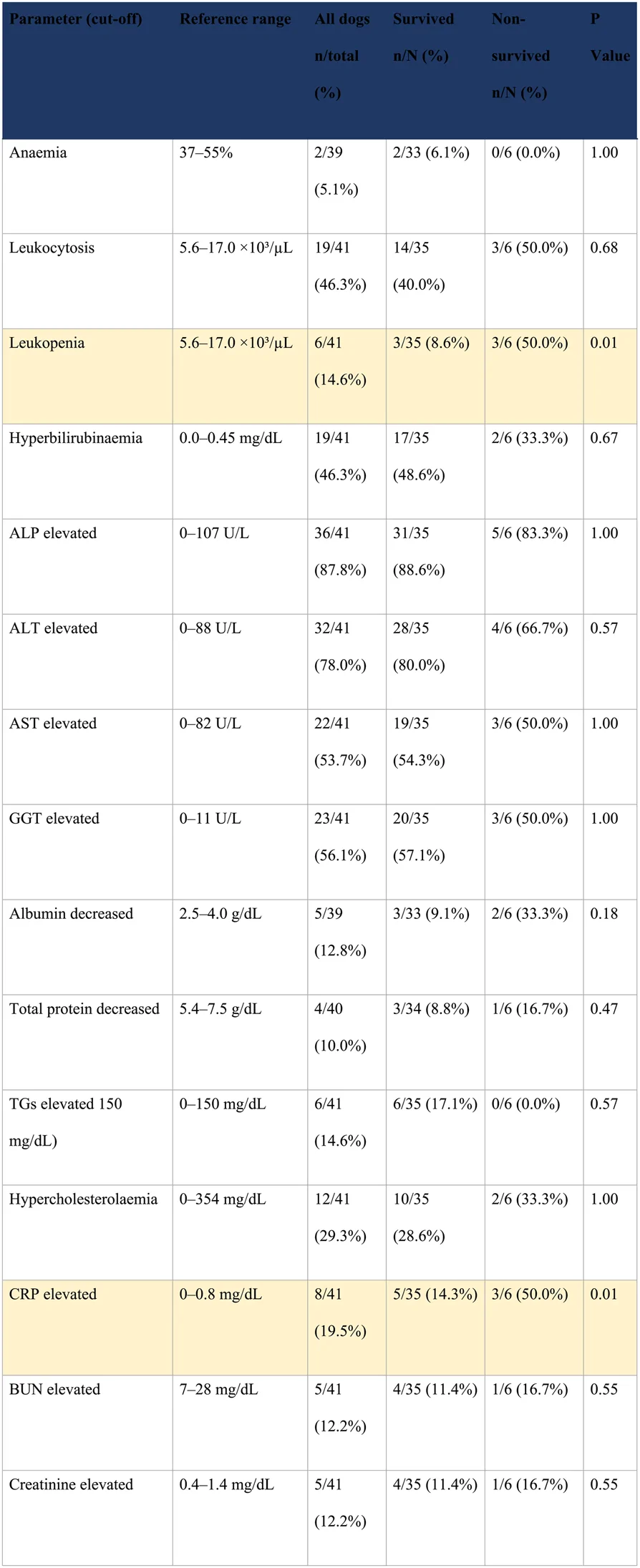
Hematobiochemical analysis evaluated in the included patients

Reference intervals are those used at the authors’ institution for adult dogs. Not all parameters were available for all dogs; denominators reflect available data. *P*-values from Fisher’s exact test. Highlighted rows (yellow) indicate statistically significant predictors of 30-day mortality (*P* ≤ 0.05)

*ALT* alanine aminotransferase, *AST* aspartate aminotransferase, *GGT* gamma-glutamyl transferase, *CRP* C-reactive protein, *BUN* blood urea nitrogen, *PCV* packed cell volume, *TGs* triglycerides

Concurrent diseases were identified in 16 dogs (39%), with 7 dogs (17.1%) presenting with two or more concurrent conditions (Table 2). These included myxomatous mitral valve degeneration (3/41, 7.3%), hyperadrenocorticism (6/41, 14.6%), diabetes mellitus (2/41, 4.9%), neoplasms (5/41, 12.2%), prostatic cysts (2/41, 4.9%), and chronic kidney disease (1/41, 2.4%).

**Table 2:** *P*-values from Fisher’s exact test comparing dogs with vs. without each concurrent disease against 30-day mortality. Neoplasia subtypes are listed individually for descriptive purposes. Prostatic cysts were identified in 2 intact male dogs. No individual concurrent disease was significantly associated with 30-day mortality (all *P* > 0.05)

Concurrent disease	*n*/total (%)	*P*-value (vs.30 day mortality)
Hyperadrenocorticism (Cushing’s syndrome)	6/41 (14.6%)	1
Neoplasia*	5/41 (12.2%)	0.82
Myxomatous mitral valve degeneration	3/41 (7.3%)	0.58
Diabetes mellitus	2/41 (4.9%)	1
Prostatic cysts	2/41 (4.9%)	1
Chronic kidney disease	1/41 (2.4%)	1
Any concurrent disease	16/41 (39.0%)	0.68

*1 Mast cell tumor, 2 Adrenal mass, 1 Pituitary  adenoma and 1 Malignant Melanoma

All 41 dogs underwent cholecystectomy via midline celiotomy. Intraoperative macroscopic evidence of GBM was confirmed in all cases. In the five cases with ultrasonographic suspect of biliary tree rupture, the surgical exploration confirmed the complete lesion of the gallbladder. Catheterisation of the common bile duct was performed in 25 dogs (61%) based on ultrasonographic findings suggestive of biliary tract dilation. Normograde catheterisation was performed in 20 dogs (48.8%), while retrograde catheterisation via duodenotomy was performed in 5 dogs (12.2%). Concurrent surgical procedures were performed in 29 dogs (70.7%) and included Jackson-Pratt drain placement in 3 dogs (7.3%), and hepatic lobectomy in 2 dogs (4.9%). Concurrent liver biopsy was performed in 29 dogs (70.7%) at discretion of the attending surgeon, based on preoperative or intraoperative evidence of hepatic abnormalities (e.g., increased echogenicity, or elevated hepatic enzymes, or gross discolouration, irregular surface), in order to obtain additional diagnostic information regarding liver pathological conditions and function that could influence postoperative treatment and prognosis. The median duration of surgery was 50 min (range, 45–76.2 min). Intraoperative complications occurred in 8 dogs (19.5%) and included hemorrhage requiring additional hemostatic measures in 3 dogs (7.3%), hypotension in 3 dogs (7.3%), hypothermia in 2 dogs (4.9%), hypertension (mean arterial pressure > 180 mmHg for > 5 min) and bradycardia (< 60–80 bpm) in 1 dog (2.4%) respectively.

Bile samples were collected for aerobic bacterial culture and antimicrobial sensitivity testing in 40 of 41 cases (97.6%). Anaerobic testing was not available. Bacterial growth was detected in 8 samples (20%), with negative cultures in 32 samples (80%). Of the 8 culture-positive samples, Escherichia coli was the most frequently isolated organism (4/8, 50.0%), followed by Serratia species, Streptococcus species, Klebsiella species, and Pseudomonas species, each identified in 1 sample (12.5% each). Histology confirmed presence of cystic mucinous hyperplasia due to GBM in all 41 cases. Additional histopathological findings included chronic cholecystitis in 9 dogs (22%), cholecystitis with necrosis in 10 dogs (24.4%), and cholecystitis with focal necrosis and ulceration in 5 dogs (12.2%). In the remaining 17 dogs (41.5%), no significant inflammatory changes were identified. Regarding liver biopsy (29 dogs, 70.7%), histology revealed moderate hepatitis in 9 cases (31%), lymphoplasmacytic hepatitis in 5 (17.2%), lymphoplasmacytic hepatitis with chronic neutrophilic infiltration in 4 (13.8%), necrotizing hepatitis in 4 (13.8%), and unremarkable findings in the remaining 7 dogs (24.1%).

Postoperative complications occurred in 2 dogs (4.9%): bile leakage at the common bile duct following retrograde catheterisation via duodenotomy, requiring revision surgery in 1 dog (2.4%), and acute kidney injury requiring intensive management in 1 dog (2.4%). The median duration of hospitalization for dogs surviving to discharge was 3 days (range 1–9 days). All dogs that survived to discharge (35/41, 85.4%) completed the 30-day follow-up period. In-hospital death occurred in 3 dogs (7.3%): cardiopulmonary arrest in 2 dogs (4.9%) and aspiration pneumonia in 1 dog (2.4%). A further 3 dogs (7.3%) died or were euthanized within 30 days from surgery following discharge. Causes included diabetic ketoacidosis in 1 dog (euthanasia at day 8), likely representing perioperative decompensation of pre-existing diabetes mellitus; autoimmune haemolytic anaemia in 1 dog (euthanasia at day 9), a condition not directly attributable to the surgical procedure; and suspected pulmonary thromboembolism in 1 dog (day 18), which may have been related to a pre-existing hypercoagulable state rather than the surgical intervention per se. The overall 30-day mortality rate was 14.6% (6/41 dogs).

Statistical analysis identified four significant predictors of 30-day mortality: elevated CRP (> 0.8 mg/dL, *P* = 0.01), preoperative leukopenia (*P* = 0.01), abdominal effusion (*P* = 0.05), and necrotizing cholecystitis (*P* = 0.05) (Table 3). Elevated CRP was present in 3/6 non-surviving dogs (50.0%) compared to 7/35 surviving dogs (20.0%). Leukopenia was observed in 3/6 non-survivors (50.0%) versus 9/35 survivors (25.7%). Abdominal effusion was present in 5/6 non-survivors (83.3%) and 26/35 survivors (74.3%). Necrotizing cholecystitis was identified in 3/6 non-surviving dogs (50.0%) compared to 7/35 survivors (20.0%).

**Table 3:** *P*-values from Fisher’s exact test

Predictor	Timing of availability	Survived 30d *n*/*N* (%)	Died 30d *n*/*N* (%)	*P*-value
Preoperative predictors				
Leukopenia	Preoperative CBC	3/35 (8.6%)	3/6 (50.0%)	0.01*
CRP elevated	Preoperative biochemistry	5/35 (14.3%)	3/6 (50.0%)	0.01*
Abdominal effusion	Preoperative ultrasound	26/35 (74.3%)	5/6 (83.3%)	0.05*
Histopathological predictor (postoperative)				
Necrotizing cholecystitis	Post-surgical histopathology (days–weeks)	7/35 (20.0%)	3/6 (50.0%)	0.05*

Preoperative predictors (leukopenia, elevated CRP, abdominal effusion) are clinically available before or at the time of surgery and can inform perioperative risk stratification. Necrotizing cholecystitis is a histopathological diagnosis available only several days after surgery and therefore cannot be used for preoperative or early postoperative decision-making; it represents an interesting prognostic observation that warrants further investigation

*Statistically significant (*P* ≤ 0.05)

## Discussion

Small-breed dogs were predominant in this cohort, with a median weight of 11.5 kg, consistent with previous reports [1, 7, 25]. Clinical presentation was nonspecific, with lethargy, vomiting, abdominal pain, and inappetence being the most common signs [7, 26]. Among the 3 dogs with clinical icterus, serum total bilirubin ranged from 5.23 to 9.00 mg/dL (median 6.50 mg/dL), well above the threshold typically associated with visible icterus in dogs (> 1.5–2.0 mg/dL). In contrast, the majority of dogs in this cohort had bilirubin values below this threshold despite frequent ultrasonographic evidence of biliary tract dilation (61%), suggesting that obstruction was incomplete or early-stage in most cases.

Abdominal ultrasound proved valuable for identifying surgical candidates. Ultrasonographic findings suggestive of peritonitis and abdominal effusion were important diagnostic indicators, though systematic analysis of abdominal fluid was not performed in all cases. The lack of correlation between Choi’s classification and patient’s outcome in the present paper is in line with the literature [24]. For this reason, since the correlation between ultrasonographic patterns of GBM and the clinical outcome remains debated, surgical decisions should be based on a comprehensive assessment of clinical, laboratory, and imaging findings rather than ultrasonographic classification alone [24, 26, 27].

Catheterisation of the common bile duct was performed in 25 dogs (61%) based on individual operator assessment, considering clinical presentation, ultrasonographic findings suggestive of biliary tract dilation, intraoperative evaluation, and degree of hyperbilirubinaemia — with markedly elevated serum bilirubin representing an additional factor favouring catheterisation in selected cases. This procedure was not significantly associated with outcome (*P* > 0.05); however, one case of retrograde catheterisation resulted in common bile duct leakage requiring revision surgery. Comparative data on dehiscence or leakage rates between normograde and retrograde techniques remain limited in the veterinary literature [16, 17]. This complication, along with previous reports of increased risk of pancreatitis and gastrointestinal complications [16, 17], supports a selective application of common bile duct catheterisation, limiting its use to cases with clear evidence of extrahepatic biliary obstruction, markedly elevated bilirubin, or compelling intraoperative findings.

Concurrent diseases were identified in 39% of cases, reflecting the typical profile of dogs affected by GBM, which are often elderly animals with underlying systemic conditions. The most frequently observed comorbidities in this cohort were hyperadrenocorticism (14.6%), neoplasia (12.2%), and diabetes mellitus (4.9%), consistent with previously reported associations between GBM and endocrine or metabolic dysfunction [2, 4–6]. Hypothyroidism, also reported in the literature as a predisposing condition, was not identified in our cohort. Although these conditions have the potential to influence the perioperative and postoperative outcome, statistical analysis did not reveal a significant association between these concurrent diseases and the 30-day mortality rate (*P* > 0.05). This finding suggests that, in appropriately selected and stabilized patients, the presence of concurrent disease does not preclude successful surgical management of GBM.

Hypercholesterolemia was present in 29% of cases, consistent with previous reports which couple metabolic alterations to GBM [4]. Elevated CRP (> 0.8 mg/dL) was the most significant biochemical predictor of outcome, present in 3/6 non-surviving dogs (50.0%) compared to 7/35 surviving dogs (20.0%; *P* = 0.01). This is in line with previous studies which identified CRP as a valuable inflammatory marker in both human and veterinary medicine for predicting complications in biliary disease [22, 23, 28, 29]. Leukopenia emerged as a critical prognostic factor, observed in 3/6 non-surviving dogs (50.0%) versus 9/35 surviving dogs (25.7%; *P* = 0.01). In human medicine, leukopenia is recognized in acute cholecystitis as a marker of severe systemic illness with a mortality rate up to 44% [30, 31]. These deaths often result from systemic complications rather than the biliary condition itself. The parallel findings in our canine population suggest that leukopenic patients face significantly increased postoperative morbidity and mortality, and their prognosis should be considered more guarded.

The 30-day mortality rate of 14.6% in this study aligns with recent reports [10, 18, 20]. When considering only deaths directly attributable to surgical complications or failure to recover from anaesthesia, the surgery-specific in-hospital mortality rate was 7.3% (3/41), more consistent with recently reported figures for elective cholecystectomy [12, 18]. The three post-discharge deaths were attributable to complications of concurrent systemic disease rather than to the surgical procedure itself. A poorer outcome was associated with necrotizing cholecystitis (documented by the serious histopathological findings), leukopenia and abdominal effusion at preoperative ultrasonography. Leukopenia and abdominal effusion, being preoperatively detectable, may serve as early indicators of disease severity [7, 27]. Liver biopsy (performed in 70.7% of cases) confirmed the frequent coexistence of hepatic parenchymal disease in this population. This was consistent with previous reports linking chronic biliary obstruction to secondary hepatic injury [32].

This study has several limitations. First, both the retrospective nature of the study and the limited sample size reduce the statistical power of the results. Moreover, the lack of systematic abdominal fluid analysis when effusion was present may have limited our ability to distinguish between septic and non-septic peritonitis, which could have provided additional prognostic information. Additionally, testing for anaerobic bacteria in bile cultures was not available at our institution, thus potentially underestimating the true prevalence of bacterial infections. Finally, the variability in surgical decision-making, particularly regarding the common bile duct catheterisation, reflects an individual operator decision rather than standardized criteria, which may have influenced the outcome. Therefore, being a single-center study, the results may not be fully generalizable, though the consistency of the surgical and anesthetic protocols within our center provided procedural standardization that may have reduced this variability. Additionally, a potential selection bias should be acknowledged: dogs with more severe disease presentation, such as confirmed gallbladder rupture, marked systemic illness, or haemodynamic instability, may have been less likely to undergo surgery, either because owners declined treatment or because the anaesthetic risk was deemed prohibitive. Financial constraints inherent to veterinary practice may have further contributed to case selection bias, resulting in a study population potentially skewed towards less critically ill patients and an underestimation of true mortality risk in the broader clinical population. Ultimately, prospective multicenter studies with a larger sample of dogs and standardized protocols are needed to validate these findings.

In conclusion, leukopenia, elevated CRP, and abdominal effusion are preoperatively identifiable predictors of poor outcome in dogs undergoing cholecystectomy for GBM, and their early recognition is essential for risk stratification and perioperative decision-making. High-risk patients identified on the basis of these parameters may benefit from aggressive preoperative stabilisation and careful perioperative monitoring. Necrotizing cholecystitis was also identified as a histopathological predictor of mortality; as histopathological results are typically available only several days after surgery, this finding cannot meaningfully contribute to postoperative management decisions, but represents an interesting prognostic observation that warrants further investigation. Common bile duct catheterisation should be performed selectively in cases with clear evidence of extrahepatic biliary obstruction. The presence of concurrent diseases in appropriately selected patients does not preclude successful surgical management, though a comprehensive perioperative care is essential.

## Data Availability

The datasets used and/or analysed during the current study are not publicly available but are available from the corresponding author on reasonable request.
